# Hyaluronic acid methacrylate/laponite hydrogel loaded with BMP4 and maintaining its bioactivity for scar-free wound healing

**DOI:** 10.1093/rb/rbad023

**Published:** 2023-03-22

**Authors:** Likun Chang, Yulong Xu, Zhouying Wu, Yichun Shao, Dan Yu, Wenyue Yang, Liyuan Ye, Xinyu Wang, Binbin Li, Yixia Yin

**Affiliations:** State Key Laboratory of Advanced Technology for Materials Synthesis and Processing, Wuhan University of Technology, Wuhan 430070, China; State Key Laboratory of Advanced Technology for Materials Synthesis and Processing, Wuhan University of Technology, Wuhan 430070, China; State Key Laboratory of Advanced Technology for Materials Synthesis and Processing, Wuhan University of Technology, Wuhan 430070, China; State Key Laboratory of Advanced Technology for Materials Synthesis and Processing, Wuhan University of Technology, Wuhan 430070, China; State Key Laboratory of Advanced Technology for Materials Synthesis and Processing, Wuhan University of Technology, Wuhan 430070, China; State Key Laboratory of Advanced Technology for Materials Synthesis and Processing, Wuhan University of Technology, Wuhan 430070, China; State Key Laboratory of Advanced Technology for Materials Synthesis and Processing, Wuhan University of Technology, Wuhan 430070, China; State Key Laboratory of Advanced Technology for Materials Synthesis and Processing, Wuhan University of Technology, Wuhan 430070, China; State Key Laboratory of Advanced Technology for Materials Synthesis and Processing, Wuhan University of Technology, Wuhan 430070, China; State Key Laboratory of Advanced Technology for Materials Synthesis and Processing, Wuhan University of Technology, Wuhan 430070, China

**Keywords:** HAMA/Lap/BMP4, photocurable nanocomposite hydrogel, sustained release, scar-free wound healing

## Abstract

Scar-free wound healing is a challenging process due to the excessive deposition of extracellular matrix and collagen. To overcome this issue, hydrogels with superior biochemical and mechanical properties have been used in combination with medicinal compounds as wound dressings. In this study, a novel composite hydrogel consisting of double-crosslinked photocurable hyaluronic acid methacrylate (HAMA) and Laponite (Lap) loaded with bioactive bone morphogenetic protein 4 (BMP4) was developed and thoroughly characterized for its properties such as degradation, morphology, porosity, compression, skin adhesion and load release. The effect of the HAMA/Lap/BMP4 hydrogel was evaluated through both *in vitro* and *in vivo* experiments. In the *in vivo* rabbit ear-scar model, the HAMA/Lap/BMP4 hydrogel dressing was found to reduce scar-related expressions of α-SAM and decrease the ratio of collagen Ι/III in wounded tissue. Additionally, histopathological examination indicated that the HAMA/Lap/BMP4 hydrogel-treated groups exhibited enhanced wound repair and increased levels of collagen maintenance compared to other standard groups, ultimately leading to scarless wound healing. Therefore, this sustained-release photocurable HAMA/Lap/BMP4 hydrogel offers a therapeutic approach for scar-free wound healing.

## Graphical Abstract

**Figure:**
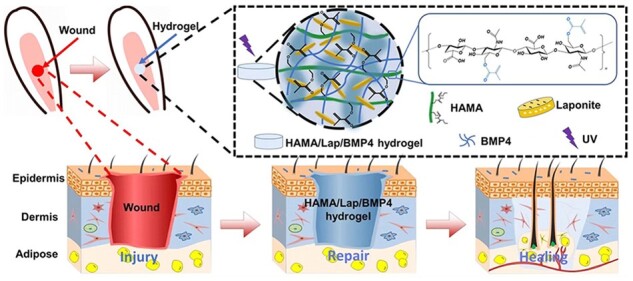

## Introduction

The skin, the largest organ in the body, serves various functions. Scarring is a common consequence of skin damage, resulting from excessive collagen deposition and the growth of myofibroblasts, collectively known as scars [1]. Current clinical and non-surgical methods have limitations such as subsequent secondary trauma and side effects may be caused, and thus, there is a pressing need to find a biomaterial with good therapeutic effects, and the ability to repair scars quickly. Biopolymer-based wound dressings and bioengineered skin substitutes, particularly hydrogels, have been widely used in wound healing due to their similarity to the extracellular matrix (ECM). Hyaluronic acid (HA), a natural polymer, is commonly used in hydrogel preparation due to its good biocompatibility, biodegradability [2] and high-water retention rate [3]. However, the fluidity of HA alone limits its application in wound healing [4]. To address this, a novel composite hydrogel consisting of double-crosslinked photocurable hyaluronic acid methacrylate (HAMA) and laponite (Lap) loaded with bone morphogenetic protein 4 (BMP4) was developed to improve the mechanical strength, drug loading and biological activity of the hydrogel. Lap has been employed as an inorganic crosslinking agent to prepare nanocomposite hydrogels with the capacity for prolonged drug release [5], and BMP4 has been shown to stimulate the differentiation of fat cells [6–8] and inhibit collagen formation and myofibroblast proliferation during the healing process.

**Figure 1.**
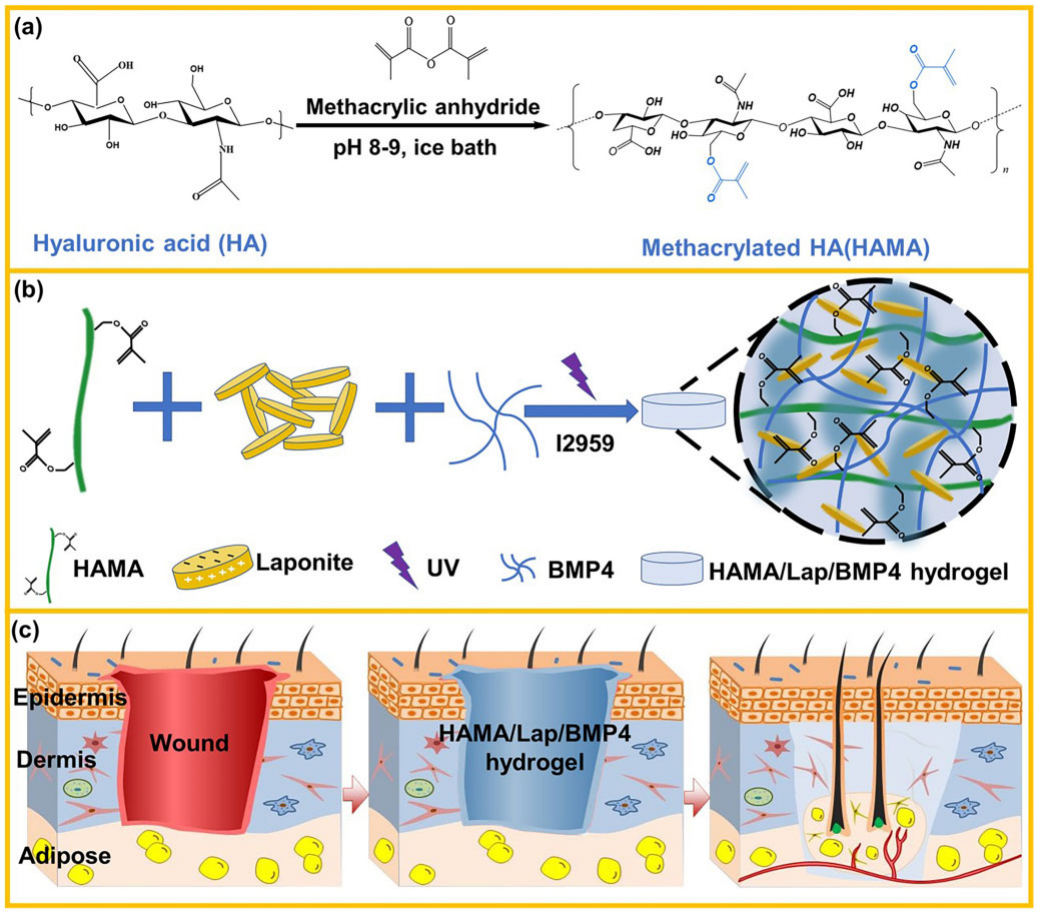
Schematic diagram of hyaluronic acid methacrylate/laponite hydrogel loaded with BMP4 and its promotion of scar-free wound healing. (**a**) The synthesis route of Hama. (**b**) Precursor solutions were prepared and shaped under UV light. (**c**) The HAMA/Lap/BMP4 composite hydrogel was covered on the surface to achieve scar-free wound healing.

In this study, the HAMA/Lap ratio was optimized to provide a material with improved mechanical properties, swelling properties and sustained release factors. *In vitro* cell experiments were conducted to examine the mechanism of scar-free wound healing, and *in vivo* animal scar models were used to investigate the effect of HAMA/Lap/BMP4 hydrogel on scar-free wound healing. The results indicated that the HAMA/Lap/BMP4 hydrogel dressing reduces scar-related expressions of α-SMA and decreases the ratio of collagen Ι/III in wounded tissue, leading to scarless wound healing. This novel composite hydrogel offers a promising therapeutic approach for scarless wound healing (Fig. 1).

## Experimental section

### Material

HA (Mv: 40–100 kDa) was purchased from McLean Co., Ltd, *N*,*N*-dimethylformamide (DMF), lysozyme (Lys) and photo-initiator (I2959) were obtained from Sinopagic Chemical Reagents Co., Ltd, methacrylic anhydride (MA) was purchased from Sigma Co., Ltd, and lithium magnesium silicate (Lap) was purchased from Beijing Yi Wei Chemical Technology Co., Ltd. BMP4 was obtained from Beijing Cerec Life Science Technology Co., Ltd. Phosphate-buffered saline (PBS), trypsin (0.25%), Dulbecco modified Eagles medium (DMEM, HyClone) supplemented with 10% (v/v) fetal bovine serum (FBS) and 1% (v/v) penicillin/streptomycin were purchased from Hyclone (China). Cell counting Kit-8 (CCK-8), Enhanced BCA Protein Assay kit (BCA), Calcein-AM (living cells were labeled green), PI dye (dead cells were labeled red) and hematoxylin were obtained from Servicebio (China).

### Formation of Hama polymers

The synthesis method has been reported in the literature, and the specific method was modified as follows [9]: 0.5 g of HA was dissolved in a mixture of DMF (15 ml) and water (30 ml) and stirred at a speed of 1000 rpm at room temperature. After the solution was completely dissolved, 3 ml of MA was slowly dropped and reacted in an ice bath for 24 h. The pH was adjusted to 8–9 with NaOH, then 3 times the volume of anhydrous ethanol was added for product precipitation. After precipitation, the supernatant was removed, and the precipitate was centrifuged at 10 000 rpm for 10 min in a high-speed centrifuge (HERMLEZ323K, Germany). The final precipitates were dialyzed (dialysis bag Mv = 14 000), and the water was changed three times a day for three days.

### Preparation and characterization of photocurable hydrogels

#### Preparation of photocurable HAMA/Lap and HAMA/Lap/BMP4 hydrogels

It has been reported that 0.5% of I2959 was suitable as a photo-initiator for wound dressing [10]. In simple terms, HAMA hydrogel was dissolved in I2959 to prepare HAMA (2, 4 and 6, m/v %), and then, different concentrations of Lap (0.5, 1.25, 2, m/v%) were added into HAMA solution and mixed evenly to obtain HAMA/Lap prepolymer. BMP4 powder was prepared into a solution of 10 μg ml^−1^ with a protein dilution buffer, diluted and blended with HAMA/Lap prepolymer. The final concentration of BMP4 was 1 μg ml^−1^, and HAMA/Lap/BMP4 pre-gel was obtained. The pre-gel was poured into the plastic mold and exposed to ultraviolet radiation (7.2 mV cm^−2^, 365 nm) for a specific time (30, 40 and 50 s) to form the hydrogel. Supplementary Table S1 listed nine orthogonal experimental groups with different HAMA, Lap concentration and UV exposure times.

#### Nuclear magnetic hydrogen spectrum

The functional degree of HAMA was determined by ^1^H-NMR. In simple terms, HAMA was dissolved in Deuterium oxide (D_2_O) of 10 mg ml^−1^ and transferred to the nuclear magnetic detection tube. ^1^H NMR spectrum was adopted by a nuclear magnetic hydrogen spectrometer (Bruker, Germany), and the frequency was 600 MHz at room temperature.

#### Fourier transform infrared spectroscopy

Three milligrams of HAMA hydrogel was weighed and fully ground with dried potassium bromide crystals. The samples were then prepared on a platen and transferred to a Fourier transform infrared spectrometer (Nexus, Thermo Nicolet, USA) for testing.

#### Swelling performance

In order to simulate osmotic pressure under physiological conditions *in vivo*, PBS buffer was used to measure the swelling rate of the hydrogel. The dried hydrogel (0.1 g ml^−1^, w/v) was immersed in PBS and shaken at 120 rpm and 37°C. The expanded weight was extracted from the solution at a specific time and measured. The weights of hydrogels after gelation (*W*_e_) and lyophilization (*W*_d_) were measured. The swelling ratio of hydrogels is calculated using Equation (1).


(1) $$\mathrm{Swelling}\;\left(\%\right)=\frac{W_{e}-W_{d}}{W_{e}}\times 100\%$$

#### Mechanical properties

The compression strength of the hydrogel was measured by a universal testing machine (Instron Corporation, USA) at a constant speed of 1 mm min^−1^ without preloading. Cylindrical hydrogels 8 mm in height and 8 mm in diameter were freshly prepared for testing. The corresponding compression modulus was calculated according to the linear interval of the obtained stress–strain curve. Meanwhile, when the stress–strain curve drops sharply, the failure stress is obtained from the curve point. Three samples were tested, and the average value of each group was calculated. The modulus (*E*) was determined from the compression stress and strain curves using Equation (2).


(2) $$E=\frac{\sigma_{2}-\sigma_{1}}{\varepsilon_{2}-\varepsilon_{1}}$$

where *σ* is the stress and *ε* is the strain.

#### Degradation performance

Freeze-dried hydrogels are weighed and then immersed in wound simulation solution (consists of a mixture of NaCl, Cacl_2_·2H_2_O and H_2_O), and oscillated at a speed of 100 rpm at 37°C. The hydrogel was extracted from the solution at a specific time (*t*), washed with deionized water, and then freeze-dried. The remaining weight at each time point was calculated, and the degradation was assessed using Equation (3). where *W*_0_ is the initial weight of hydrogel after freeze-drying and *W_t_* is the weight of hydrogel at the time point (*t*) after freeze-drying.


(3) $$\mathrm{Degradation}\;\left(\%\right)=\frac{W_{0}-W_{t}}{W_{0}}\times 100\%$$

#### Morphology and porosity

Hydrogel morphology was observed by field emission scanning electron microscope (JSM-7500F, JEOL). The freeze-dried hydrogel samples were spray-coated with a thin gold film to observe the morphology of the hydrogel, and the porosity was determined by ImageJ software.

#### Skin adhesion test

After forming the hydrogel in the mold, a quick preliminary test was performed by checking the adhesion of the hydrogels on human fingers. Then, the adhesion of hydrogel to pig skin was explored [11] (Fig. 3c and d). Firstly, fresh pork skin was cut into 76 mm by 25 mm strips. Due to pig skin’s opacity, a slide of the same size was coated with gelatin 25 mm^2^ to simulate pig skin tissue. Then the freshly prepared HAMA pre-gel and HAMA/Lap composite hydrogel were uniformly dropped on top of the gelatin. The skin sample covered the hydrogel, reaching a bonding area of 25 mm^2^. Then, the sandwiched structure was turned over and photocured [12]. Finally, the bond strength was measured using a universal testing machine at a crosshead velocity of 5 mm min^−1^. Three samples were tested, and the average value of each group was calculated.

#### Simulated release

An appropriate amount of hydrogel loaded with lysozyme was weighed and immersed in 3 ml of PBS (pH = 7.4), respectively, in a 37°C shaking table for simulated factor release, and 300 μl of PBS was aspirated at 1, 3, 6, 12, 24, 48 and 72 h, respectively, and 300 μl of fresh sterile PBS was timely added into the centrifuge tube. The collected samples were stored in a −20°C freezer for testing. The concentration of PBS for each time period (*C*_t_) was calculated by using the standard curve measured at 562 nm with the BCA kit (Servicebio, China), and the cumulative release (*M*_t_) was calculated according to the following Equation (4).


(4) $$M_{t}=C_{t}\times 3+\sum C_{t-1}\times 0.3$$

### *In vitro* biological evaluation of hydrogels

#### Blood compatibility

The hemolysis activity of the hydrogel was measured with red blood cells (RBC) from healthy rabbits. An appropriate amount of hydrogel was introduced into 1.5 ml centrifuge tubes, 1 ml of RBC solution was added to each tube and incubated at 37°C for 3 h. All samples were centrifuged at 2000 rpm for 10 min, and the absorbance of the supernatant was measured at 540 nm by the enzyme-linked immunoassay (Multiskan, Thermo Scientific). The hemolysis rate was calculated using Equation (5).


(5) $$\mathrm{HR}\;\left(\%\right)=\frac{A_{s}-A_{n}}{A_{p}-A_{n}}\times 100\%$$

where *A*_s_, *A*_p_ and *A*_n_ were sample absorbance, positive control (distilled water), negative control (normal saline) and absorbances, respectively.

#### Cell proliferation detection

L929 fibroblast (Type Culture Collection of the Wuhan University, Wuhan, China) suspension was inoculated in 96-well plates with a density of 4 × 10^4^ cells ml^−1^. When the cells adhered to the well, the supernatant was aspirated, and 0.1 g ml^−1^ (m/v) material extract was added to the well plate. CCK-8 (Servicebio, China) was added to the test well on the first, third and fifth days and incubated for 2 h in an incubator with 5% CO_2_ at 37°C, protected from light, and the absorbance was measured at 450 nm. There were six parallel samples in each group. Cell viability was calculated by Equation (6).


(6) $$\mathrm{Cell}\;\mathrm{viability}\;\left(\%\right)=\frac{\mathrm{OD}-\mathrm{Blank}}{\mathrm{Control}-\mathrm{Blank}}\times 100\%$$

where OD is the absorbance of the extract, Control is the absorbance of the cell group only, and Blank is the absorbance of the medium group only.

#### Cell activity test

To further observe cell activity, NIH/3T3 cell (Type Culture Collection of the Wuhan University, Wuhan, China) suspensions were inoculated on 6-well plates for live/dead staining. At the specified time point, the cells were stained with 2 μM Calcein-AM (living cells were labeled green) and PI dye (dead cells were labeled red) (Servicebio, China) for 25 min. The cell morphology was observed under an inverted fluorescence microscope (Olympus IX71, Japan), and the cell survival rate was assessed by ImageJ software. All experiments were repeated three times.

#### Wound healing experiment

Scratch experiment was carried out to study the effect of hydrogel on the migration of fibroblasts, NIH/3T3 cells were cultured in HAMA and HAMA/Lap hydrogel-coated 6-well plates for 24 h with 2 × 10^6^ cells per well. After removing the medium, the tip of a 200 μl pipette was used to scratch the surface of the cell layer to form a cell-free area. After 24 h culture, the exfoliated cells were gently washed with PBS, and the hydrogel extract of each group was added to the well plate and cultured in an incubator at 37°C and 5% CO_2_ for 24 h. The cells were observed under an inverted microscope, and images were taken to monitor the migration of cells at the scratch site. The healing ratio was determined by ImageJ software [13] as Equation (7).


(7) $$\mathrm{Healing}\;\mathrm{ratio}\;\left(\%\right)=\frac{A_{0}-A_{t}}{A_{0}}\times 100\%$$

*A*_0_ and *A_t_* are the cell-free areas at 0 and 24 h, respectively.

#### Cell adhesion detection

To observe the morphology of cells on the hydrogel surface, NIH/3T3 cells with a concentration of 5 × 10^3^ cells ml^−1^ were inoculated on the hydrogel surface and cultured in a 24-well plate. After 12 h incubation, the hydrogel was gently washed with PBS and the cells were fixed with 2.5% glutaraldehyde for 1 h. The hydrogel was thoroughly washed with PBS, then dehydrated with graded ethanol (50%, 70%, 95% and 100%) and freeze-dried for 24 h. The attachment of cells to the surface of the hydrogel was observed with a field-emission scanning electron microscope.

### *In vivo* biological evaluation of hydrogels

#### Scar animal modeling

The animal experiments involved in this experiment were carried out by ISO10993-2:2000 ‘Biological Evaluation of Medical Devices Part 2: Animal Welfare Requirements’, which conformed to ethical standards. New Zealand rabbits from Hubei Center for Disease Control and Prevention were used in this study. New Zealand rabbits (2.5 kg/rabbit, 10 weeks old, female) were selected to establish hypertrophic scar model according to the previously published scheme [14, 15]. Briefly, after the ventral skin of the rabbit ear was disinfected, chloral hydrate (0.25 g/kg) was injected intravenously into the ear margin. Four full-thickness skin defects with a diameter of 1 cm were made along the long axis in the ventral part of each rabbit ear. The whole layer of skin was excised, the pericardium was scraped away, and the cartilage was retained. The distance between each defect was more significant than 1 cm [16]. Intramuscular penicillin injections were given to each New Zealand rabbit to prevent postoperative infection. Any wounds showing signs of infection or necrosis would be excluded from the study.

#### Macro observation of scar

After establishing the rabbit ear-scar model, six New Zealand rabbits were divided into three groups: control group, HAMA/Lap hydrogel group and HAMA/Lap/BMP4 composite hydrogel group, with three parallel samples in each group. The hydrogel was changed once a day to observe the morphology and color of the rabbit ear hypertrophic scar, and the wound healing and scar hyperplasia were recorded.

To further explore the mechanism of scar-free healing, the rabbits were euthanized at the end of the experiment, and the wounds and adjacent normal skin were collected and fixed in a paraformaldehyde solution (4%, v/v).

#### Histological evaluation

The sections were successively dehydrated in xylene and absolute ethanol. Sections were stained with hematoxylin for 3–8 min and rinsed slowly with distilled water. Then it was blued back with 0.6% of ammonia, washed with running water, stained in eosin for 1–3 min, dehydrated with xylene and absolute ethanol and sealed with neutral gum after drying. The images were observed under a microscope and collected [17].

#### Masson staining

The paraffin sections were dehydrated and stained with a hematoxylin staining solution for 5–10 min. After washing thoroughly, sections were dyed with Masson spring red acid complex red solution for 5–10 min, then soaked in 2% of acetic acid solution shortly, differentiated with 1% of phosphomolybdic acid solution for 3–5 min and dyed directly with aniline blue for 5 min. Finally, they were soaked in a 2% of glacial acetic acid solution, dehydrated with absolute ethanol and xylene and sealed with neutral gum [18].

#### Immunohistochemistry staining

The paraffin sections were dehydrated and incubated with 3% of hydrogen peroxide solution for 5–10 min at room temperature. The sections were then blocked with 5–10% of normal goat serum, incubated for 10 min at room temperature and serum removed at 4°C overnight with primary antibodies of α-SMA, type Ι, type III collagen and PPARγ. The following day, sections were treated with appropriate secondary antibodies and stained with diaminobenzidine.

#### Picrosirius red staining

Deparaffinated sections and hydrate in distilled water, stained with picrosirius red for 1 h, rinsed in two changes of acetic acid solution, soaked in two changes with absolute ethanol for 2 min, soaked in two changes with xylene for 3 min and finally neutral gum seal.

#### Scar elevation index

It refers to the ratio of the thickness of the healing tissue above the cartilage surface to the thickness of normal tissue above the cartilage surface. The tissue thickness was determined using ImageJ software. Scar elevation index (SEI) = 1 indicates that the recovered tissue is highly consistent with the normal skin tissue; that is, there is no scar. SEI >1 demonstrates that the healed tissue is higher than the normal skin tissue, which means scar formation [16, 19]. SEI was calculated as Equation (8).


(8) $$\mathrm{SEI}=\frac{h}{t}\;$$

The value of *h* in this formula is the thickness of the healing tissue above the cartilage surface, the value of *t* is the thickness of normal tissue above the cartilage surface.

### Statistical analysis

Each experiment was repeated thrice at least, and data were expressed as mean±SE. *P* < 0.05 was statistically significant.

## Results and discussion

### Characterization of Hama

**Figure 2.**
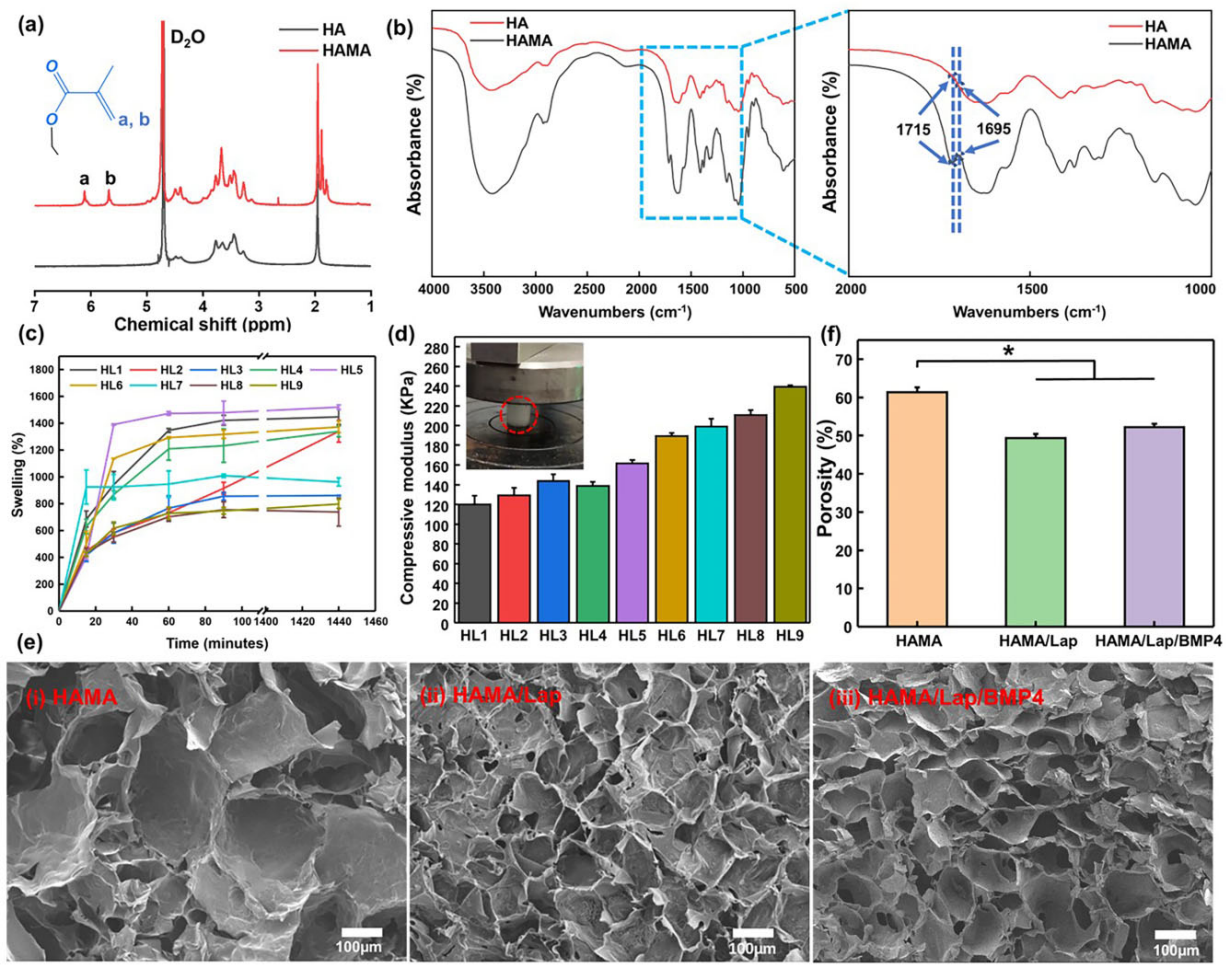
Physicochemical properties of HAMA, HAMA/Lap and HAMA/Lap/BMP4 hydrogels. (**a**) Nuclear magnetic hydrogen spectrum. (**b**) Fourier transform infrared spectroscopy. (**c**) Swelling property at different time points and (**d**) the compression modulus of orthogonal experimental group. (circle: material formed under UV light.) (**e**) Representative SEM images of lyophilized hydrogel, and (**f**) according to SEM images using ImageJ software to calculate the porosity of HAMA, HAMA/Lap and HAMA/Lap/BMP4 hydrogels (*P < 0.05, mean±SD).

HAMA was obtained by reacting MA in an ice bath with HA by activating the carbon–carbon double bond. HAMA precursor solution formed HAMA/Lap hydrogel by electrostatic adsorption to the hydrated Lap solution with the photo-initiator I2959 under UV light. Different material structures were characterized by ^1^HNMR and FT-IR. Two new peaks at ≈5.8 and ≈6.1 ppm appeared in ^1^HNMR, two asymmetric H protons in the grafted methacrylate double bond, respectively, proving the successful synthesizing of HAMA **(**Fig. 2a). In addition, the methyl peaks on the methacrylate group appeared at 1.86 and 1.79 ppm, which were quite different from the methyl peaks on the disaccharide ring [20]. Compared with the FT-IR spectra of HA, the absorption peaks at 1715 and 1659 cm^−1^ in HAMA spectra were the bending vibration peaks of the carbonyl group and double bond (Fig. 2b), which confirmed the existence of a double bond between HA and the attached methacrylate anhydride [21], which was consistent with the conclusion of ^1^H-NMR spectrum.

### Characterization of hydrogels

In the molding pre-experiment, a 1% concentration of HAMA could prepare soft hydrogels with easy deformation. However, a 7% concentration of HAMA produced hydrogels with poor fluidity was not easy to shape and possessed many bubbles that were not easy to remove. Therefore, the concentration of HAMA was selected in the range of 2–6%, while the concentration of Lap varied from 0.5% to 2% [5, 22] to generate a series of pre-gels. The illumination time was 30, 40 [4, 23] and 50 s, respectively.

In wound repair, hydrogel needs to maintain a moist environment, which is beneficial to promote the growth of wound granulation and resist bacterial invasion. The swelling ratio of the orthogonal test group of HAMA/Lap composite hydrogels (Fig. 2c) was indicated that all hydrogel groups had good water storage capacity. Within 1 h, the swelling equilibrium was reached in all other groups except for the HL2 group. Within 24 h, the swelling rates of nine orthogonal groups were more than 700%, and the swelling rate of the HL5 group was as high as 1720.59 ± 17.12%. The main reason for the difference in the swelling rate of composite hydrogels was the different pore sizes, which may be caused by different cross-linking densities, different restrictions of molecular chain movement and different water storage capacities. In addition, when a hydrogel is applied to the wound, it needs a specific deformation ability; otherwise, the hydrogel will be damaged, and the effect of wound repair will not be achieved. It has been reported that the high modulus of compression (about 100 kPa) was beneficial for the culture of keratinocytes [24]. The data (Fig. 2d) showed that the compression modulus of the nine orthogonal experimental groups were all more significant than 100 kPa. The compression modulus gradually increased with the concentration of HAMA and Lap, resulting in a more compact structure of the double-network hydrogel [25], which could have an increased resistance to the external force. According to the swelling and mechanical properties characterization results of the orthogonal experimental groups, the HL5 group (4%HAMA-1.25%Lap-50s) was selected for subsequent experiments.

The degradation performance of hydrogel is also an important index. The results (Supplementary Fig. S1) showed that the degradation rate of the two groups of hydrogels was faster at the initial stage of the experiment, and the degradation rate was about 17% before 9 h, with no significant difference. The degradation rate slowed down as time passed, reaching about 28% at 72 h. The HAMA/Lap composite hydrogel was slightly more difficult to degrade than HAMA hydrogel, which may be because HAMA/Lap hydrogel was more rigid with a smaller pore size. In this study, the content of Lap may be too small to affect the degradation performance.

HAMA, which has ample water-binding ability, was used as the substrate to facilitate the formation of porous structures during cell migration and lyophilization. Freeze-dried hydrogels were very close to their original form because no obvious deformation was observed after freeze-dried. In particular, the permeability of hydrogel is related to its internal morphology. The results of SEM (Fig. 2e) showed that the surface of all three groups of hydrogels formed a 3D porous structure. The pore size was uniform, the size was appropriate and the structure was uniform. The permeability of hydrogel could be adjusted by increasing the Lap. It facilitated the absorption of water, cell migration and fluid exchange, which were essential for supporting cell survival and growth. However, from the data (Fig. 2f), it could be seen that there was a significant difference in porosity between HAMA and that of the Lap-based hydrogels. This may be due to the loading of the Lap cross-linking agent, resulting in a dense network structure, which reduced the porosity [26] but did not affect the performance of the Lap-based hydrogels.

**Figure 3.**
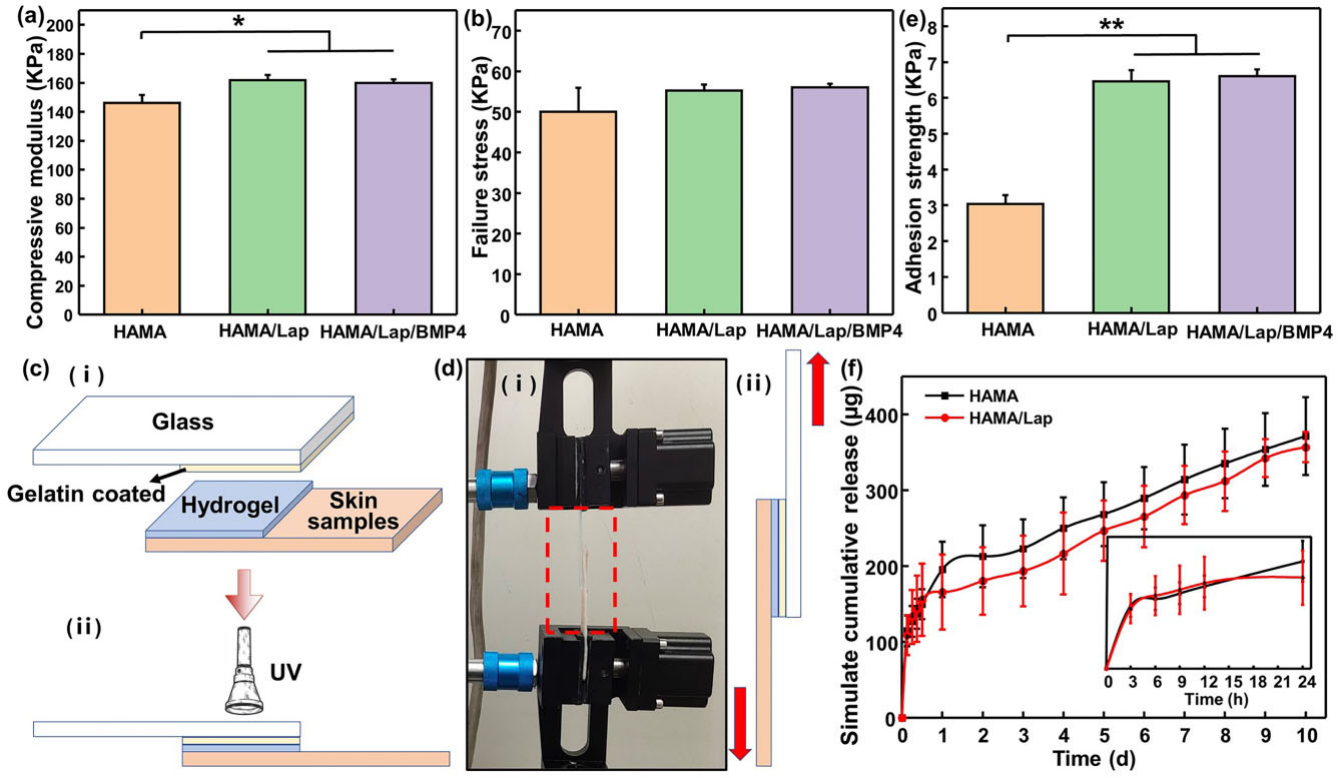
Physicochemical properties of HAMA, HAMA/Lap and HAMA/Lap/BMP4 hydrogels. (**a**) Compression modulus and (**b**) failure stress of hydrogels. (**c**) Schematic diagram of tissue adhesion. (i) The simulated samples included glass sheets with gelatin, hydrogels and pig skin. (ii) UV irradiation of hydrogel. (**d**) Simulated sample stretching. (i) Adhesion tensile front view (box: sample). (ii) Drawing diagram of the simulated sample. (**e**) Adhesion strength of hydrogels. (**f**) The cumulative release from the hydrogel within 10 days *in vitro* (the lower right corner showed the cumulative release within 1 day) (*P < 0.05, **P < 0.01, mean±SD).

Compression modulus and failure strength are the main characteristics of hydrogel resistance to deformation. The compressive modulus and the failure stress of hydrogels (Fig. 3a and b) indicated that Lap could enhance the resistance of hydrogel to deformation. This may be due to the increased cross-linking degree of hydrogel as Lap loading, the activity of the molecular chain segment was blocked and the stress required to generate unit shape variables in the deformation resistance region increased compared to pure HAMA. Therefore, HAMA/Lap composite hydrogel had a high deformation resistance ability.

Good tissue adhesion is beneficial for hydrogels to control bleeding and seal wounds effectively by adhering to tissue surfaces [27]. The adhesion of the two groups of hydrogels on fingers (Supplementary Fig. S2a) preliminarily indicated that the viscosity of HAMA/Lap composite hydrogel was stronger than HAMA hydrogel. To further explore tissue adhesion, HAMA and HAMA/Lap hydrogels were tested for their adhesion strength at two different adhesion interfaces, pig skin and gelatin-coated slides (Supplementary Fig. S2b). The bonding strength of Lap-based hydrogels were significantly higher than that of HAMA hydrogel (Fig. 3e). The experimental results confirmed that Lap improved the bonding strength of HAMA/Lap composite hydrogel. The reason for the improved cohesive strength of the hydrogel structure may be attributed to Lap’s inherent viscosity and the possible electrostatic interactions between HAMA and Lap, which enhances the crosslinking density [28, 29].

Two sets of hydrogels loaded with lysozyme were placed in PBS to simulate *in vivo* release, in which the initial burst release rates in the first 3 h were 28.63 ± 5.07% and 27.24%±6.61% and then continued to be released slowly. The cumulative release over 10 days were 92.8 ± 12.92% and 89.16 ± 4.99% (Fig. 3f), respectively. This showed that Lap could slow the release of proteins [30–33].

### *In vitro* biological evaluation of hydrogels

In HAMA and HAMA/BMP4 hydrogel groups, RBCS were clustered at the bottom, whereas RBCS in HAMA/Lap and HAMA/Lap/BMP4 hydrogel groups adhered to the surface of the hydrogel (Supplementary Fig. S3a). Hemolysis rates (Supplementary Fig. S3c) were all below the acceptable limit (5%) [34]. Therefore, all four hydrogels were wound dressings with good blood compatibility.

The results (Supplementary Fig. S3b and d) showed that, as the culture time increased, the experimental groups demonstrated sustained cell proliferation activity compared to the control group. This indicated that materials exhibited excellent cytocompatibility [35].

**Figure 4.**
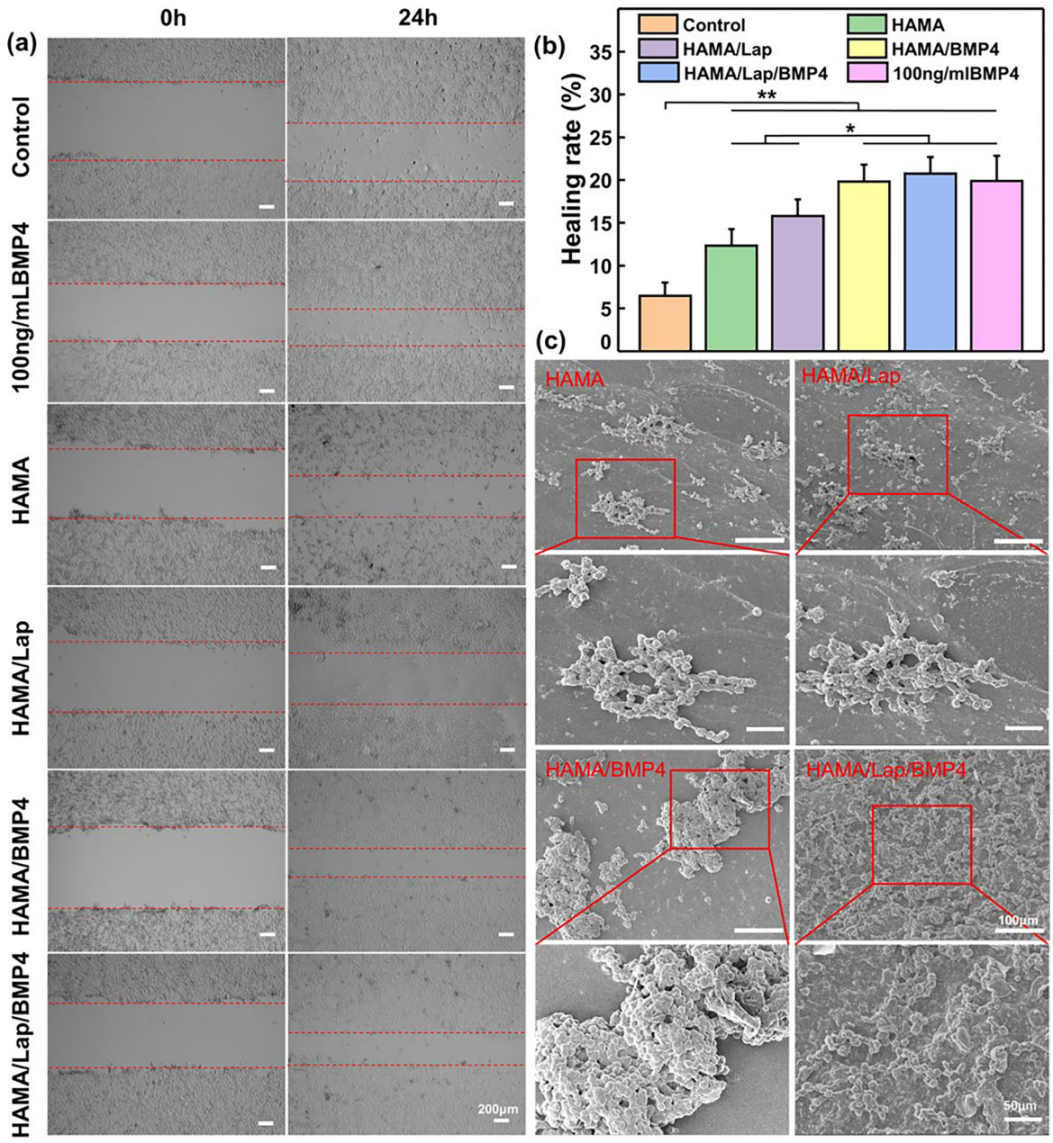
*In vitro* biological evaluation of hydrogels. (**a**) Migration images of NIH/3T3 cells under an inverted microscope for 0 and 24 h (cell-free areas in the dashed line). (**b**) Healing rates were calculated by ImageJ software. (**c**) SEM images of NIH/3T3 cells cultured in hydrogels after 12 h (*P < 0.05, **P < 0.01, mean±SD).

The scratch test can simulate wound healing *in vitro* to determine whether the hydrogel can promote cell healing [36]. NIH/3T3 migrated and covered the scratched area within 24 h (Fig. 4a). By observing and photographing scratches at different time points, the scratch widths were measured by ImageJ software and the healing rates of the scratch area were calculated. The healing rates were 6.47 ± 1.52%, 12.31 ± 1.93%, 15.81 ± 1.91%, 19.81 ± 1.96%, 20.76 ± 1.93% and 19.88 ± 2.94% in the control group, four groups of wound dressings and 100 ng ml^−1^ concentration of BMP4 group, respectively. The results showed that compared with the control group, there were significant differences in all groups except the HAMA group. Besides, there were also significant differences between the BMP4-loaded hydrogel group and the BMP4-unloaded hydrogel group. Combined with the simulated release results, the simulated release amount in 24 h was 36.82% of the total release amount, which was consistent with the trend of scratch experiment results. As could be seen from the data (Fig. 4b), the four groups of hydrogels had a faster healing rate than the control group, and the healing rate of the BMP4-loaded hydrogel group was higher, indicating that HAMA/Lap/BMP4 hydrogel could better promote cell migration.

The cell adhesion was observed by scanning electron microscopy to test the affinity of hydrogels for cells. After 12 h, hydrogels were analyzed by scanning electron microscopy (Fig. 4c), and the attachment of NIH/3T3 fibroblasts was qualitatively evaluated. The SEM images displayed that NIH/3T3 cells had attached to the surface of the hydrogel after 12 h of culture, and the cell attachment to the HAMA/Lap/BMP4 composite hydrogel was significantly higher than that of other hydrogel groups, and the adhesion was uniform. We hypothesized that due to the presence of both positive and negative charges on the Lap surface, the unique anisotropy could produce an electrostatic adsorption with both cells and BMP4 [13], resulting in increased cell adhesion, which was consistent with the results reported in the literature [37] that Lap could promote cell adhesion.

### *In vivo* biological evaluation of hydrogels

**Figure 5.**
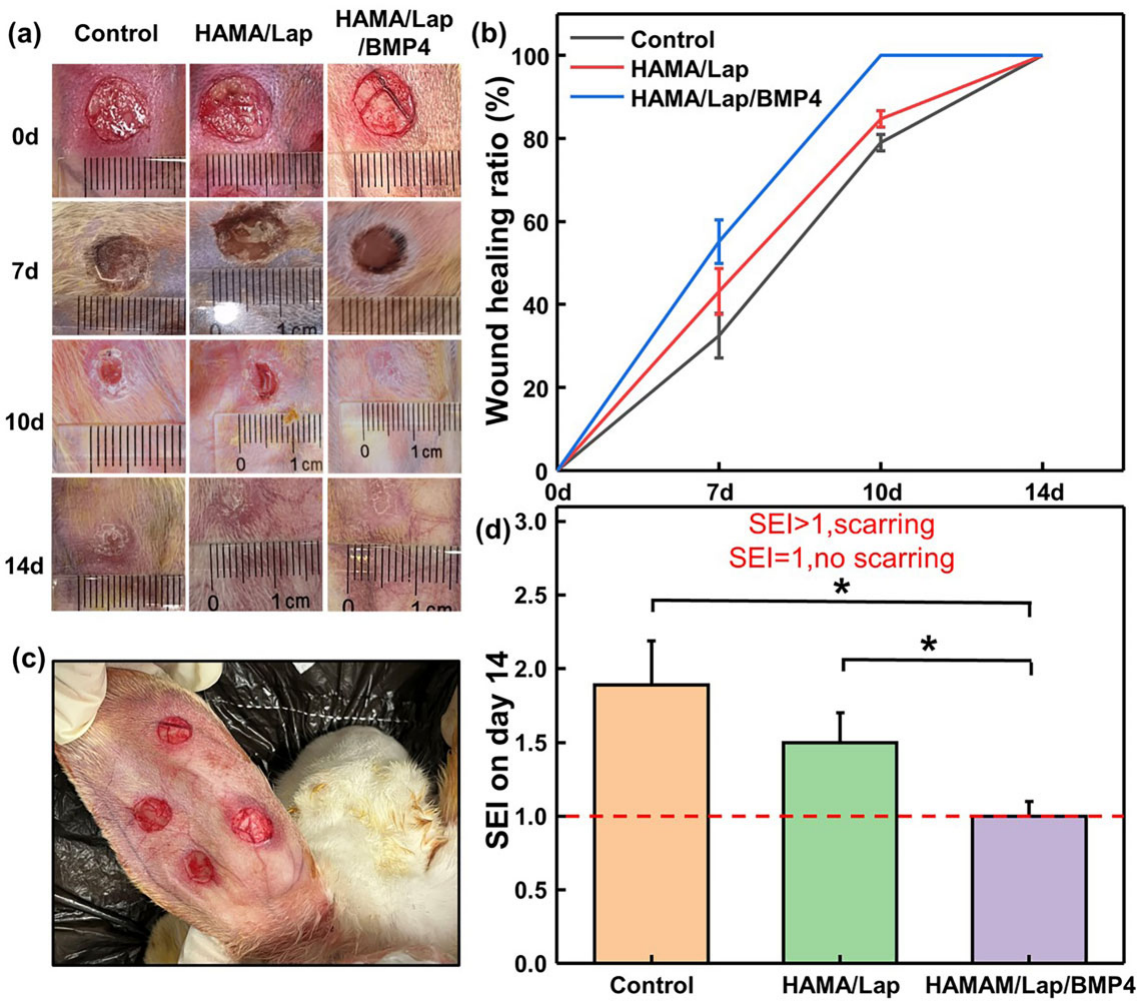
Model establishment and treatment of full-thickness skin wound of the rabbit ear. (**a**) Macro observation of wound size for 0, 7, 10 and 14 days. (**b**) Wound healing rates were calculated by ImageJ software for 7, 10 and 14 days. (**c**) Rabbit ear-scar model. (**d**) Scar elevation index (SEI) was calculated by ImageJ software for 14 days (*P < 0.05, mean±SD).

After the rabbit ear-scar model was established (Fig. 5c), the hydrogel was applied to the full-layer wound of the rabbit ear. After 2 weeks, the defect has completely healed, and a hypertrophic scar was formed. On days 0, 7, 10 and 14 after the operation, the wounds of the three groups gradually began to heal, and the HAMA/Lap/BMP4 hydrogel significantly decreased at the wound area on day 7. On the 10th day, the healing was complete, while the other groups were not completely healed, and on the 14th day, the skin tone was light, the rest of the groups skin tone was pinker [38] (Fig. 5a). The healing rates of Control, HAMA/Lap and HAMA/Lap/BMP4 hydrogel on day 7 were 32.5 ± 5.43%, 43.13 ± 5.58% and 55.14 ± 5.23%, respectively. On the 10th day, the healing rates were 79.27 ± 2.89%, 84.68 ± 1.63% and 100%, respectively. All groups had completely healed by 14 days (Fig. 5b). Compared with the control group, HAMA/Lap and HAMA/Lap/BMP4 hydrogel effectively compensated for defects during early healing in the regeneration stage, thereby promoting wound healing.

The scar height was then further observed. On the 14th day, the scar thickness of the hydrogel group was significantly lower than that of the control group (Supplementary Fig. S4). To quantitatively assess scar thickness, we measured the thickness of newly formed tissue after healing and compared it to the normal tissue thickness above the cartilage. We then calculated their ratios to determine the SEI. An SEI value greater than 1 indicates scarring, while an SEI of 1 indicates no scarring. The results (Fig. 5d) showed prominent scar bulges in the control group and a few scars in the HAMA/Lap hydrogel group. In contrast, the HAMA/Lap/BMP4 hydrogel significantly inhibited scarring after full-thickness wound healing.

**Figure 6.**
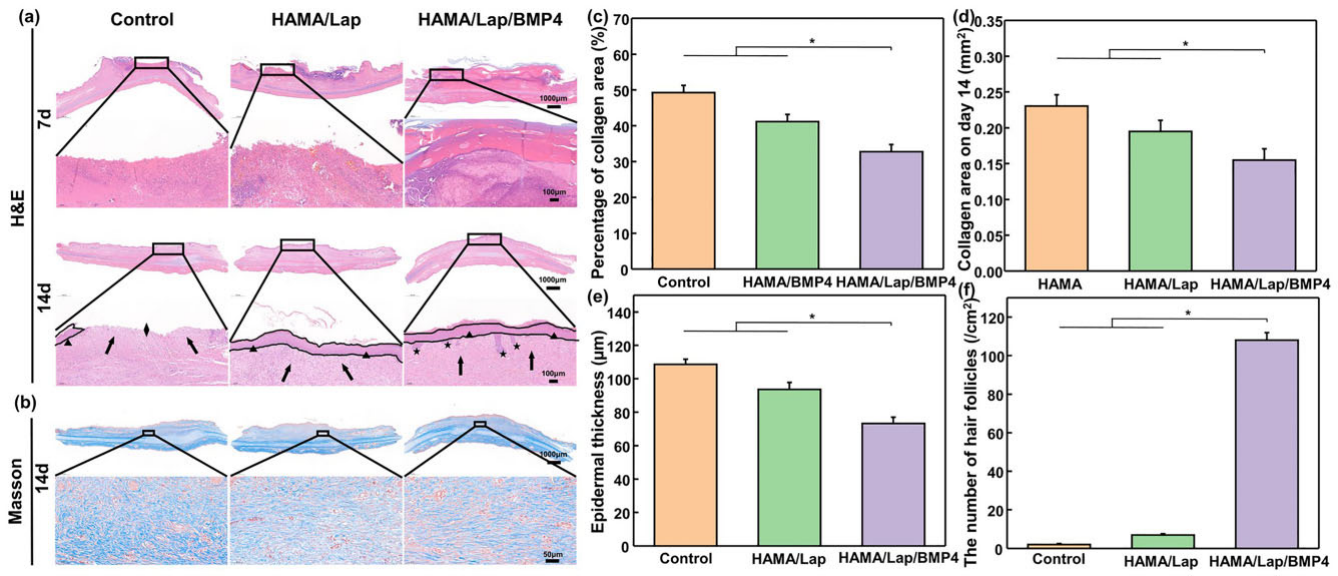
H&E and Masson staining histological analysis at different time points posttreatment. (**a**) H&E staining for 7 and 14 days (triangle: intact epidermis, diamond: incomplete epidermis, arrow: fibroblast, pentagram: skin appendage hair follicle) on day 14, (**b**) Masson staining and its (**c**) collagen area, (**d**) collagen area percentage were calculated. (**e**) Epidermal thickness and (**f**) the number of hair follicles were calculated by ImageJ software (*P < 0.05, mean±SD).

Further pathological changes, including scab, wound healing, epidermal thickness and skin appendage generation, were analyzed by H&E staining (Fig. 6a). On day 7, H&E staining images of tissue sections in all three groups showed varying degrees of inflammatory cell infiltration. Still, there were fewer inflammatory cells in HAMA/Lap/BMP4 hydrogel, and the scab was obvious in all three groups. At the same time, the H&E staining image of the wound was darker, and many lymphocytes, macrophages and epithelial cells migrated to the wound, thus causing an inflammatory reaction and entering the inflammatory stage [19, 39]. It was found that the number of inflammatory cells in the control group and the HAMA/Lap hydrogel group was significantly higher than that in the HAMA/Lap/BMP4 hydrogel group, indicating that the wound inflammation was more severe in the control group and the HAMA/Lap hydrogel group. On day 14, although the control group was not completely epithelialized, granulation tissue formation and no scab were also observed [40]. The HAMA/Lap and HAMA/Lap/BMP4 hydrogel groups formed a uniform epidermal layer, and granulation tissue and collagen fibers were formed in large amounts. In addition, the HAMA/Lap/BMP4 hydrogel group was accompanied by the generation of skin appendages such as hair follicles. The results indicated that HAMA/Lap and HAMA/Lap/BMP4 hydrogel groups had intact skin tissue on the wound surface, promoting scar-free healing.

Scar formation is generally due to excessive collagen deposition and abnormal fibrosis, resulting in a hypertrophic scar on the wound surface [41, 42]. Masson staining was further used to analyze the density and arrangement of collagen fibers. On day 7, scabs were observed in all three groups, but collagen fibers began to form in the HAMA/Lap and HAMA/Lap/BMP4 hydrogel groups (Supplementary Fig. S5). On day 14, collagen fibers in the control group had the highest density and the most disordered arrangement, presenting a vortex arrangement. In addition, we found that the thickness of collagen fibers decreased in the HAMA/Lap hydrogel group, but the arrangement of collagen fibers was still showing significant vortices. At the same time, compared with the control group and the HAMA/Lap hydrogel group, the collagen fiber density in the HAMA/Lap/BMP4 hydrogel group was lower, the collagen fiber was more orderly organized, and the gap between the collagen fiber bundles was more significant, which was parallel to the wound and neatly arranged, similar to normal skin (Fig. 6b). According to the analysis of Masson staining, the collagen area and percentage of the HAMA/Lap/BMP4 hydrogel group were significantly lower than those of the other two groups, and there was a significant difference (Fig. 6c and d). Recovery of skin wounds is accompanied by the regeneration of skin tissue and the reconstruction of related skin appendages. Therefore, we quantified the epidermal thickness [43, 44] and number of hair follicles [44, 45] of the wound skin tissue on day 14, and the results (Fig. 6e and f) showed that the HAMA/Lap and HAMA/Lap/BMP4 hydrogel groups had lower epidermal thickness and a significant increase in the number of hair follicles compared to the control group. The results confirmed that HAMA/Lap/BMP4 hydrogel reduced scar thickness by inhibiting the formation of collagen fibers in the scar.

**Figure 7.**
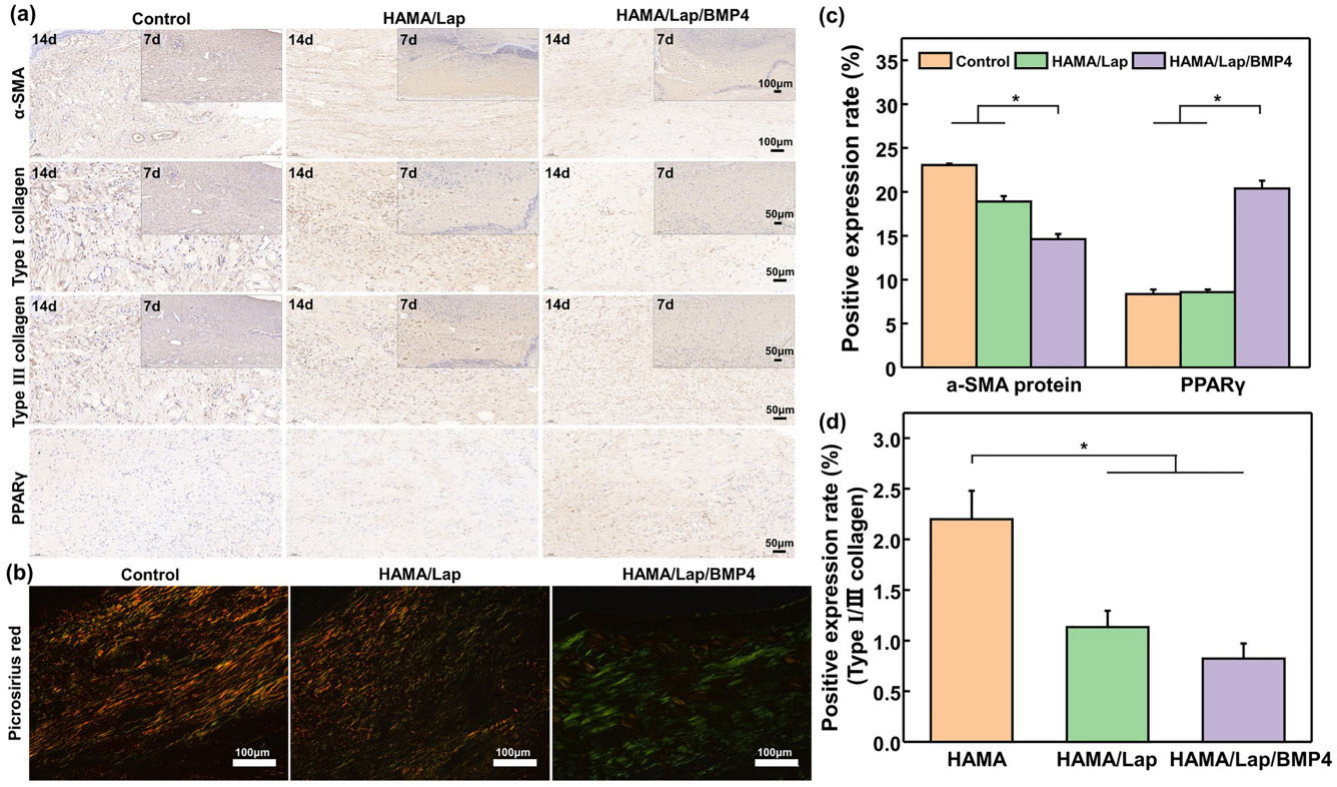
Immunohistochemical analysis posttreatment for 7 and 14 days. (**a**) α-SMA protein staining, type I, type III collagen staining for 7 and 14 days, and PPARγ staining for 14 days. (**b**) The images of picrosirius red staining under polarized light microscopy and its (**d**) ratio of type I/III collagen were counted by ImageJ software for 14 days. According to the immunohistochemical results, (**c**) the positive expression rate of α-SMA actin and PPARγ were quantified by ImageJ software for 14 days (*P < 0.05, mean±SD).

In addition, the expressions of α-SMA protein, type I [46], type III collagen and PPARγ were studied to explore further the mechanism of promoting wound healing and reducing scar. The effect of hydrogel on accelerating wound healing and inhibiting scar was investigated. During wound healing, fibroblasts proliferate and differentiate into myofibroblasts, which can not only secrete α-SMA protein to promote wound healing but also secrete type I and type III collagen for ECM reconstruction [47]. At a later stage, myofibroblasts reprogram new hair follicles to transform into fat cells [48]. In immunohistochemistry, the nucleus was stained blue and secreted markers brownish yellow (Fig. 7a). It has been reported that the expression ratio of collagen I/III in the early stage could determine the structure of granulation tissue, and the increase in the expression ratio of collagen I/III could enhance the stability of wound tissue [49]. However, in the later stage, a low ratio of collagen I/III was conducive to wound healing and scar formation, and a high value indicated excessive collagen production. Results of positive expression rates (Fig. 7c and Supplementary Fig. S6) showed that the HAMA/Lap/BMP4 hydrogel could significantly inhibit the expression of myofibroblast marker α-SMA [38] and type I collagen [50, 51] on skin wounds and improve the expression of type III collagen and fat cell markers PPARγ, indicating that it could inhibit scar formation in the healing process. The picrosirius red staining (Fig. 7b) [45] was consistent with the trend of immunohistochemistry, and the ratio of collagen I/III (Fig. 7d) was statistically counted, which showed that the HAMA/Lap/BMP4 hydrogel did inhibit the deposition of collagen. We hypothesized that fibroblasts over-proliferate during wound healing into myofibroblasts, and collagen over-deposition forms scar. However, after injection of the hydrogel at the wound site, the transdifferentiated myofibroblasts reprogram the new hair follicles to activate the BMP4/Smad pathway, thereby regenerating fat cells and inhibiting scar formation [47, 48].

## Conclusion

In this study, a novel double cross-linking HAMA/Lap/BMP4 composite hydrogel was successfully prepared to for scar-free wound healing. The hydrogel was formed by the electrostatic interaction between negatively charged HAMA and anisotropic crosslinking agent Lap, followed by loading BMP4 as a carrier, and photo-crosslinked. The hydrogel gradually released BMP4, promoting wound healing. The physical and chemical characteristics of the HAMA/Lap composite hydrogel showed better wound healing, exudate absorption and resistance to deformation. *In vivo* results showed a decrease in scar formation by reducing α-SMA protein production and the fraction of I/III collagen, increasing the expression of the marker PPARγ in adipocytes. The HAMA/Lap/BMP4 composite hydrogel also exhibited good biocompatibility, promoting cell adhesion and migration. Therefore, the HAMA/Lap/BMP4 composite hydrogel has the potential to prevent scar formation and stimulate the growth of skin appendages such as hair follicles.

## Supplementary Material

rbad023_Supplementary_Data
