# Supplementary material for: Hyaluronic acid methacrylate/laponite hydrogel loaded with BMP4 and maintaining its bioactivity for scar-free wound healing
Source: Regen Biomater. 2023 Mar 22;10:rbad023. doi: 10.1093/rb/rbad023 (PMC10081883; doi:10.1093/rb/rbad023)
Supplement: rbad023_Supplementary_Data [file rbad023_supplementary_data.pdf]

Supporting Information

Hyaluronic acid methacrylate/Laponite hydrogel loaded with BMP4 and maintaining its bioactivity for scar-free wound healing

Likun Chang, Yulong Xu, Zhouying Wu, Yichun Shao, Dan Yu, Wenyue Yang, Liyuan Ye, Xinyu Wang, Binbin Li, Yixia Yin*

State Key Laboratory of Advanced Technology for Materials Synthesis and Processing, Wuhan University of Technology, Wuhan, 430070, China

*Correspondence address. Tel: 13657219796; E-mail: yinyixia@whut.edu.cn (Y.Y.)

These authors contributed equally to this work.

Content:

Table S1. Optimization of HAMA/Lap hydrogels.

Figure S1. Degradation rate at different times.

Figure S2. Finger adhesion and tissue adhesion diagram.

Figure S3. In vitro biological evaluation of hydrogels.

Figure S4. Macroscopic observation of the scar on day 14.

Figure S5. Masson staining images of the three hydrogels on day 14.

Figure S6. Quantitatively analyze the positive expression rate of (a) type I collagen and (d) type III collagen at different time points by Image J software.

Table S1. Orthogonal experimental group. The influencing factors included HAMA, Lap concentration and UV exposure time.

Group	HAMA	Laponite	UV duration
HL1	2%	0.5%	30s
HL2	2%	1.25%	40s
HL3	2%	2%	50s
HL4	4%	0.5%	40s
HL5	4%	1.25%	50s
HL6	4%	2%	30s
HL7	6%	0.5%	50s
HL8	6%	1.25%	30s
HL9	6%	2%	40s

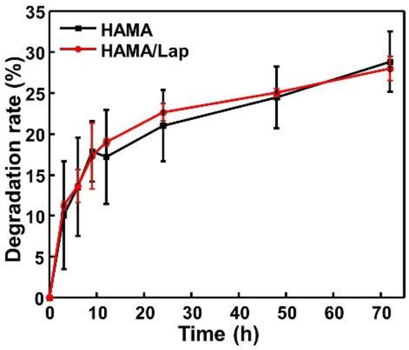

Figure S1. Degradation rate at different times.

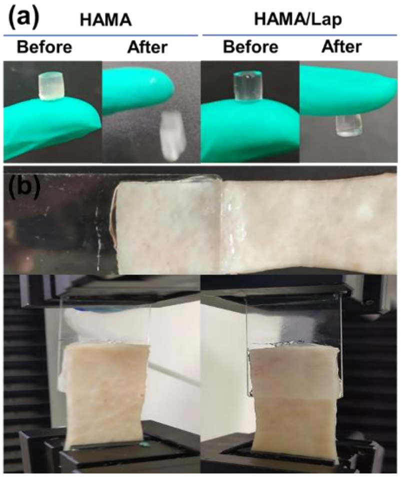

Figure S2. Finger adhesion and tissue adhesion diagram. (a) Image of hydrogel sticking to finger and inverted. (b) Front and side view of test sample.

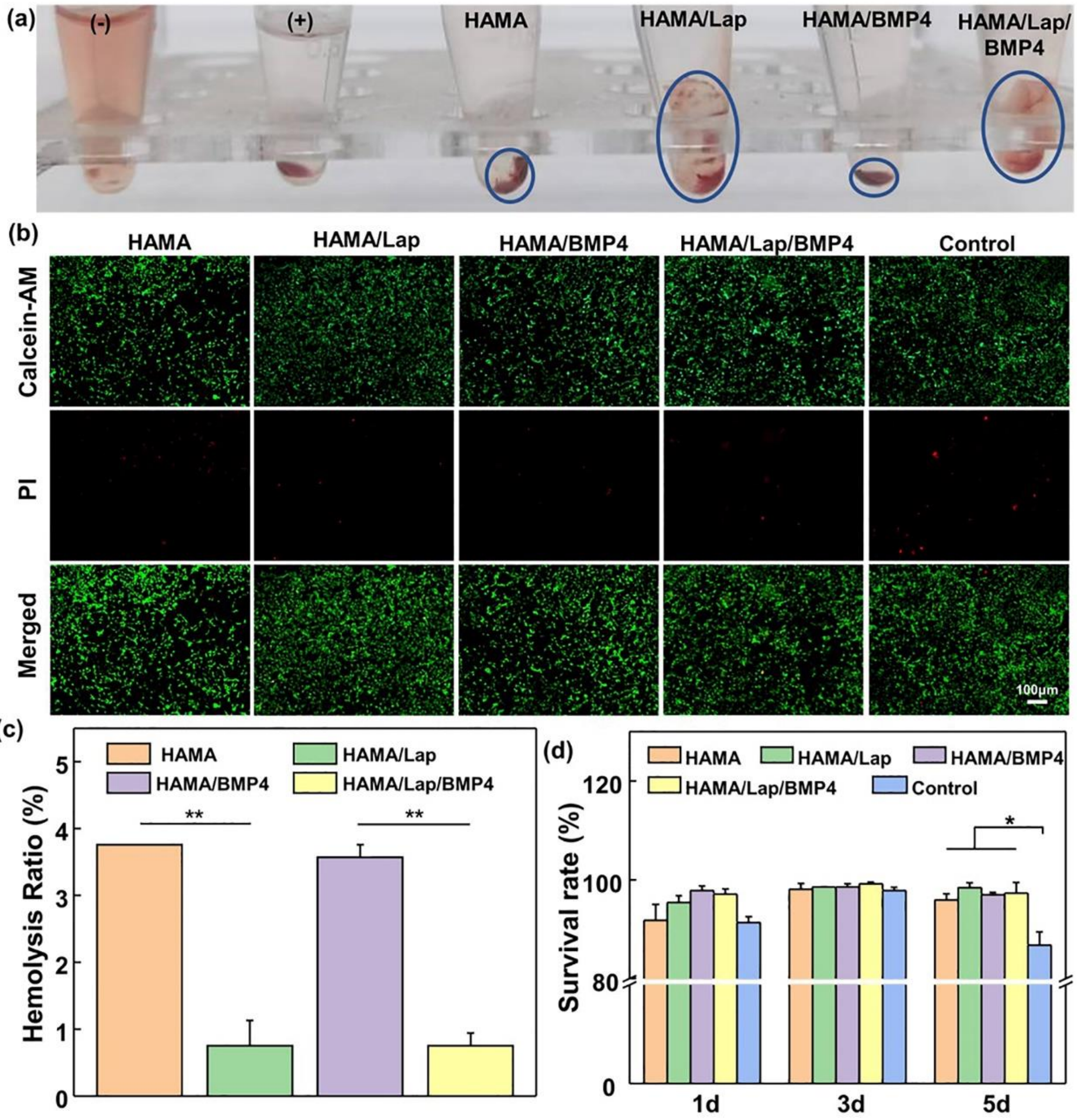

Figure S3. In vitro biological evaluation of hydrogels. (a) Hemolysis (negative control was deionized water, positive control was normal saline. Circles indicate the distribution of red blood cells on the hydrogel). (b) NIH/3T3 cell viability on day 5 (living cells stained green, dead cells stained red). (c) Hemolysis rate calculated by ImageJ software. (d) NIH/3T3 cell viability on day 1, 3, and 5 (control group: no material). (*$p < 0.05$, **$p<0.01$, mean ± SD.)

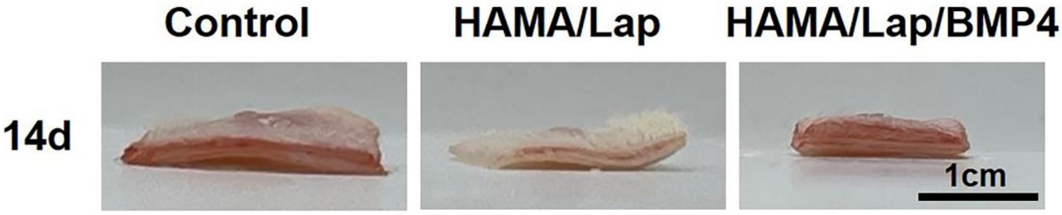

Figure S4. Macroscopic observation of the scar on day 14.

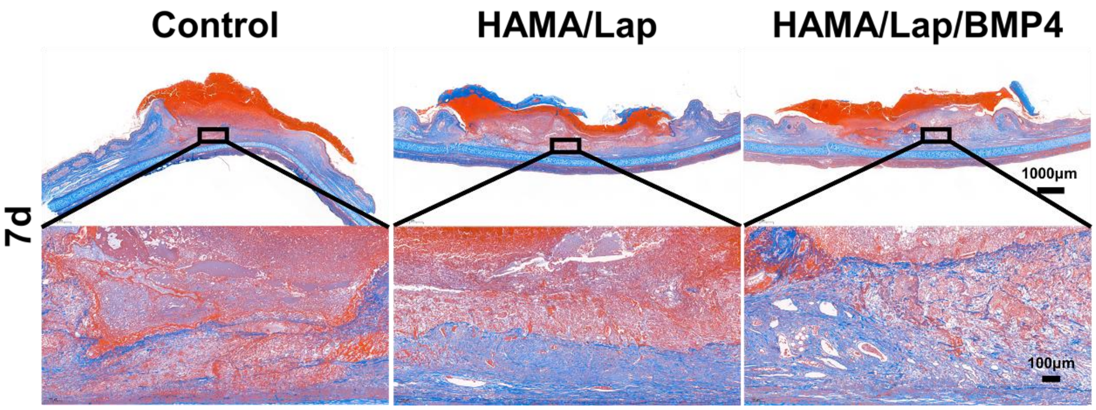

Figure S5. Masson staining images of the three hydrogels on day 14.

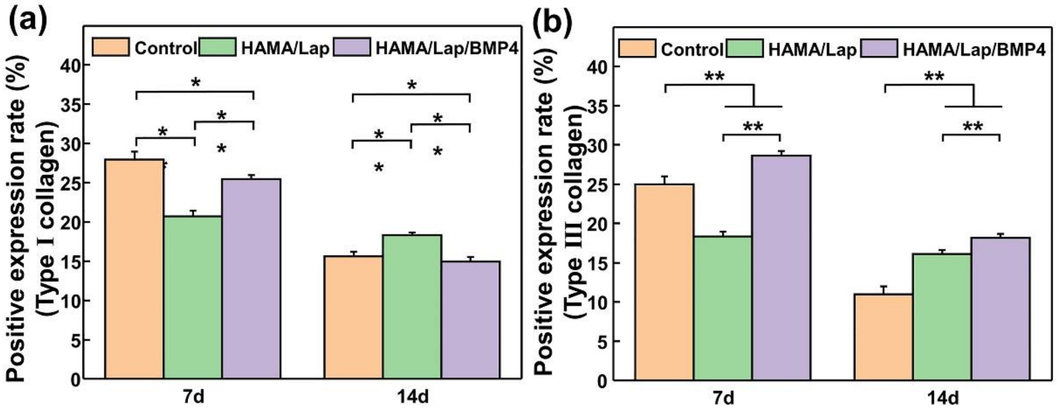

Figure S6. Quantitatively analyze the positive expression rate of (a) type I collagen and (d) type III collagen at different time points by Image J software. (*p < 0.05, **p<0.01, mean ± SD.)
